# Treatment of the lung injury of drowning: a systematic review

**DOI:** 10.1186/s13054-021-03687-2

**Published:** 2021-07-19

**Authors:** Ogilvie Thom, Kym Roberts, Susan Devine, Peter A. Leggat, Richard C. Franklin

**Affiliations:** 1 College of Public Health, Medical and Veterinary Sciences, James Cook University, Townsville, QLD Australia; 2 Department of Emergency Medicine, Sunshine Coast Hospital and Health Service, Sunshine Coast, QLD Australia; 3 School of Medicine, National University of Ireland Galway, Galway, Ireland; 4 Royal Life Saving – Australia, National Office, Broadway, Sydney, Australia

**Keywords:** Drowning, Review, Ventilation, Non-invasive ventilation, Lung injury

## Abstract

**Background:**

Drowning is a cause of significant global mortality. The mechanism of injury involves inhalation of water, lung injury and hypoxia. This systematic review addressed the following question: In drowning patients with lung injury, what is the evidence from primary studies regarding treatment strategies and subsequent patient outcomes?

**Methods:**

The search strategy utilised PRISMA guidelines. Databases searched were MEDLINE, EMBASE, CINAHL, Web of Science and SCOPUS. There were no restrictions on publication date or age of participants. Quality of evidence was evaluated using GRADE methodology.

**Results:**

Forty-one papers were included. The quality of evidence was very low. Seventeen papers addressed the lung injury of drowning in their research question and 24 had less specific research questions, however included relevant outcome data. There were 21 studies regarding extra-corporeal life support, 14 papers covering the theme of ventilation strategies, 14 addressed antibiotic use, seven papers addressed steroid use and five studies investigating diuretic use. There were no clinical trials. One retrospective comparison of therapeutic strategies was found. There was insufficient evidence to make recommendations as to best practice when supplemental oxygen alone is insufficient. Mechanical ventilation is associated with barotrauma in drowning patients, but the evidence predates the practice of lung protective ventilation. There was insufficient evidence to make recommendations regarding adjuvant therapies.

**Conclusions:**

Treating the lung injury of drowning has a limited evidentiary basis. There is an urgent need for comparative studies of therapeutic strategies in drowning.

**Supplementary Information:**

The online version contains supplementary material available at 10.1186/s13054-021-03687-2.

## Background

Drowning is a major cause of preventable death and morbidity worldwide. There are over 295,000 unintentional drowning deaths (excluding boating) per year [1, 2]. Ninety percent of these deaths occur in middle- and low-income countries, and half the fatalities are aged less than 25 years [1]. Despite the modern medical literature on drowning reaching back at least as far as *The Lancet* in 1878 [3], there have only recently been efforts to standardise definitions [4] and data collection [5] for drowning research. The majority of the published studies focus on three themes—preventative strategies such as secure pool fencing, high-risk groups such as children and factors determining clinical outcome, especially duration of immersion [2, 6–8].

The mechanism of drowning involves aspiration of water into the lung which damages surfactant, disrupts the alveolar capillary membrane and leads to the development of alveolar oedema, resulting in a local acute respiratory distress syndrome (ARDS)-like syndrome [6]. A high proportion of drowning patients are hypoxic and have a PaO_2_/FiO_2_ ratio < 300 mm Hg [9, 10]. Treating this lung injury and reversing the hypoxia are the cornerstone of the management of drowning [7].

However, current ventilation guidelines for drowning patients are adapted from ARDS [7, 11] and as such may not reflect the needs of the drowned patient. The aim of this paper is to review the existing evidence to guide the clinician in the treatment of the lung injury and respiratory distress associated with drowning.

## Methods

### Research question

The patient, intervention, comparison and outcome (PICO) question being addressed is: In drowning patients with lung injury, what is the evidence from primary studies regarding comparisons of treatment strategies and subsequent patient outcomes?

This is a systematic review using the Preferred Reporting Items for Systematic Reviews and Meta-Analyses (PRISMA) [12] to explore the treatment of drowning.

### Protocol

The protocol for this review is available on Prospero; “Treating the respiratory impairment of drowning: A systematic review” (CRD420203896).

### Inclusion criteria

This review included papers with human participants, who had drowned, and that included outcome data for interventions designed to treat the lung injuries associated with drowning. Outcomes of interest were mortality, escalation of ventilation strategy, duration of ventilation, ARDS, pneumonia and barotrauma. There was no restriction on publication date or age of participants. Systematic reviews and meta-analyses, where available, were included if they reported primary data outcome of interest.

### Exclusion criteria

Papers in a language other than English and animal studies were excluded. Letters, editorials, reviews and case reports were also excluded. Studies contributing data to included systematic reviews or meta-analyses were not individually included in this review.

### Search strategy

**Fig. 1:**
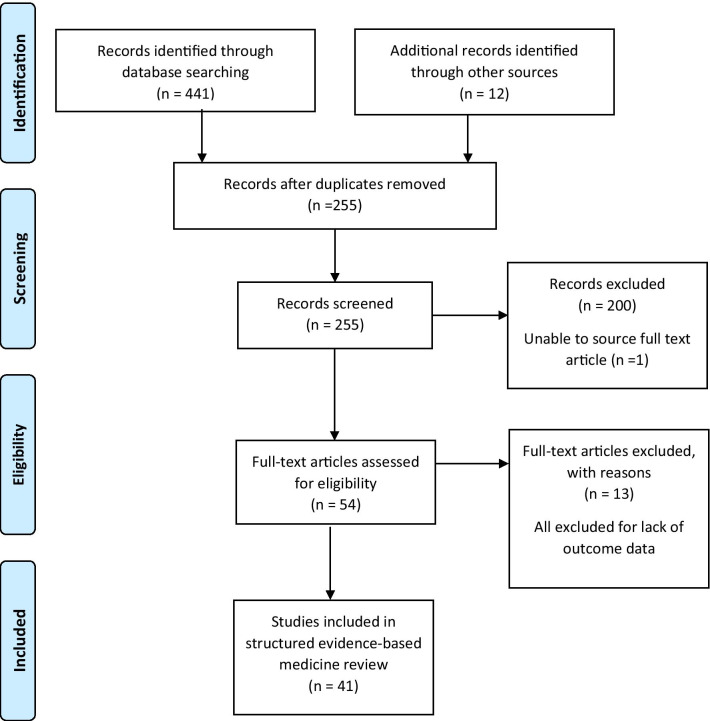
PRISMA flowchart

The PRISMA methodology for searching the literature was utilised to ensure a systematic approach was taken [12]. The search strategy was constructed for use on Medline and adapted for use on EMBASE, CINAHL, Web of Science and SCOPUS. Searches were conducted on 15 January 2021, with no date limitations. The full search strategy is detailed in Additional file 1 and included MeSH terms for the environment such “critical care” and “emergency department”, the condition “drowning” and “near drowning” and the intervention such as “non-invasive ventilation”, “mechanical ventilation” and “ECLS treatment”. Reference lists of included articles and relevant reviews were also searched. Screening of the search results by title, abstract and then full text was conducted by two authors (OT and KR) for inclusion. Where agreement was not achieved, these were referred to a third author (RF). Where outcome data were unclear, attempts were made to contact the corresponding author for clarification. The results of the search strategy are presented in Fig. 1.

### Appraisal of selected studies

Data were independently extracted from selected articles using a standardised form by two authors (OT and KR). All papers were assessed for the quality of the evidence utilising Grading of Recommendations Assessment, Development and Evaluation (GRADE) methodology [13]. Observational studies were defined prior to assessment as having a low quality of evidence [14]. The GRADE evidence profile for included studies is included in Additional file 2.

## Results

There were 41 studies which met the inclusion criteria. The summary table of included studies is detailed in Additional file 3. They included data on patients from 20 countries with the USA [15–27] and France [28–31] most frequently represented. Patient data were reported on 1973 patients. Patient demographics were incomplete with regards to gender in eleven papers [19, 20, 25–27, 31–35]. Data presented included 1093 (55.4%) males and 545 (27.6%) females and were similarly incomplete with regards to age groups in seven papers [10, 16, 31, 32, 34, 36, 37], with a minimum of 675 (34.5%) children included. Studies were predominately (30/41, 73.2%) from intensive (critical) care units [10, 16, 20–31, 34–51], 6/41 (14.6%) from inpatient units [9, 15, 17, 18, 41, 52] and five (12.2%) studies were based in the Emergency Department [19, 32, 33, 53, 54]. Three studies utilised the Utstein Style for drowning [28, 29, 44].

A total of 17 papers were identified where the lung injury of drowning was the focus of the research question [9, 10, 20–22, 28–31, 36, 37, 39, 43, 44, 47, 50, 51, 53]. Twenty-four papers where the lung injury of drowning was not the focus of the research question that nevertheless included information relevant to the review were included [15–19, 23–27, 32–35, 37, 38, 40, 41, 45, 46, 48, 49, 52, 54]. All were case series other than one retrospective cohort study [29] and two multicentre registry studies [21, 26]. The GRADE level was universally (41/41, 100%) rated as very low.

Mortality was reported in all papers. There was considerable overlap amongst treatment groups and reported outcomes frequently included multiple treatment groups. Insufficient studies were free of these issues to allow meta-analysis. Extracorporeal life support (ECLS) was the most common theme (21 articles) [20, 21, 23–27, 31, 34, 35, 37, 43–51] followed by ventilatory strategies (14 articles) [9, 10, 15, 17, 19, 28, 29, 32, 38–41, 53, 54], the use of prophylactic antibiotics to prevent aspiration or early onset post-drowning pneumonia (14 articles) [9, 10, 16–18, 30, 33, 36, 38–41], the use of corticosteroids (7 articles) [16, 18, 33, 36, 40, 41, 52] and the use of diuretics (5 articles) [9, 18, 40, 41, 52]. A summary of the aims, the population studied, the study setting, treatment strategies, methodology, results and GRADE level of the selected studies is included in Additional file 3.

### Extra-corporeal life support

The ECLS studies fall into two categories. Six studies [21, 25–27, 37, 47] report on ECLS in drowning with a survival rate of 156/290 (53.7%). Fifteen studies reported on ECLS for drowning associated with accidental hypothermia [20, 22–24, 31, 34, 35, 43–46, 48–51]. The survival rate was 35/120 (29.2%). Overall, the survival rate for ECLS in drowning is 191/410 (46.6%) (Table 1).

**Table 1:** ECLS and drowning

Study	Outcome measured	Outcome	Confounders
*Drowning*			
Steiner et al. [25]	Mortality	3/8 (37.5%) survived	No validated assessment tool used for neurological outcome
Weber et al. [27]	Mortality Neurological outcome	1/4 (25.0%) survived	No validated assessment tool used for neurological outcome
Kim et al. [47]	Mortality Neurological outcome	8/9 (88.9%) rapidly worsening ARDS 7/9 (77.8%) survived 7/9 (77.8%) good neurological outcome	No validated assessment tool used for neurological outcome
Burke et al. [21]	Mortality	60/84 (71.2%) survived (no cardiac arrest prior to ECLS) 49/86 (56.9%) survived (cardiac arrest followed by ROSC prior to ECLS) 18/77 (23.3%) survived (ECPR)	No neurologic outcome reported
Watson et al. [26]	Mortality	3/4 (75%) survival	No neurologic outcome reported
Lee et al. [37]	Mortality	15/18 (83.3%)	No neurologic outcome reported
*Drowning* + *Hypothermia*			
Saltiel et al. [22]	Mortality Neurological outcome	2/3 (66.6%) survived, 1/3 (33.3%) GNO	No validated assessment tool used for neurological outcome
Walpoth et al. [34]	Mortality Neurological outcome	0/2 (0.0%) survived	No validated assessment tool used for neurological outcome
Mair et al. [48]	Mortality Neurological outcome	1/7 (14.3%) survived	No validated assessment tool used for neurological outcome
Farstad et al. [45]	Mortality	1/14 (7%) survived	Neurological data presented as group results
Wollenek et al. [51]	Mortality Neurological outcome	2/3 (66.6%) survived 1/3 (33.3%) poor neurological outcome 1/3 (33.3%) good neurological outcome	No validated assessment tool used for neurological outcome
Eich et al. [44]	Mortality Neurological outcome USFD	5/12 (42%) survived 2/12 (17%) full recovery (PCPC 1) 3/12 (25%) (PCPC 5)	Paediatric cerebral performance category
Scaife et al. [23]	Mortality Neurological outcome	1/5 (20%) survived	No validated assessment tool used for neurological outcome
Coskun et al. [43]	Mortality Neurological outcome	5/13 (38%) survived 3/13 (23%) severe neurological deficit	No validated assessment tool used for neurological outcome
Suominen et al. [49]	Mortality Neurological outcome	1/9 (11%) survived	No validated assessment tool used for neurological outcome
Wanscher et al. [35]	Mortality Neurological outcome	7/7 (100%) survived GOSE ranged from 3–7	Group results given for neurological outcome
Skarda et al. [24]	Mortality Neurological outcome	0/7 (0%) survived	
Champigneulle et al. [31]	Mortality Neurological outcome USFD	2/20 (10%) survived 1 good neurological outcome (CPC 1) 1 severe cerebral disability (CPC 3)	Validated neurological outcome score used
Weuster et al. [50]	Mortality Neurological outcome Drowning definition	2/9 (22%) survived 2/9 GNO	No validated assessment tool used for neurological outcome
Khorsandi et al. [46]	Mortality	3/4 survived	No neurologic outcome reported
Bauman et al. [20]	Mortality Neurological outcome	3/5 survived	No validated assessment tool used for neurological outcome

ECLS, Extra-corporeal life support; GNO, good neurological outcome; ROSC, return of spontaneous circulation; ECPR, ECLS-assisted cardio-pulmonary resuscitation; PCPC, paediatric cerebral performance category; GOSE, Glasgow Outcome Scale Extended; USFD, Utstein Style for Drowning; CPC, cerebral performance category

Burke et al. published data on 247 drowning patients on the Extracorporeal Life Support Organization international database, covering a 30-year period. They reported good outcomes in patients who had not experienced cardiac arrest where ECLS was initiated for refractory respiratory failure (60/84, 71.4% survival). In post-cardiac arrest patients, where ECLS was initiated following ROSC, survival was still high but lower (49/86, 57.0%). Survival in drowning patients was lowest when ECLS was initiated during cardiac arrest (18/77, 23.4%).

### Mechanical ventilation

Mechanical ventilation (MV) is often used when supplemental oxygen alone is insufficient [15, 17, 19, 29, 39, 41, 54]. Other indications include decreased conscious state [28, 52] or cardiac arrest [15, 32, 39, 41, 54]. Complications of MV include the development of pneumonia [36] and barotrauma [15, 17, 19]. The reported frequency of barotrauma is high, with an incidence of 75%, 12% and 10% [15, 17, 19], respectively. Since these earlier papers, MV strategies have evolved into the practice termed lung protective ventilation (LPV), which decreases ventilator-associated lung injury [55], and this practice is currently advocated for the treatment of drowning patients [7, 11]. Michelet et al. reports using LPV in 30 drowning patients with no barotrauma reported [28]. Unfortunately, barotrauma was not identified as an outcome of interest in the paper. Duration of mechanical ventilation has not changed greatly. A study of 25 patients reported a mean duration of MV of 4.3 days in 1982 [17], but data from two recent papers [28, 29] demonstrate a mean (SD) duration of 6 (± 12) days (*n* = 70).

Other outcomes reported for MV typically include survival and neurological status [15, 32, 41]. Given the co-existence of hypoxic encephalopathy in many of these patients [38], it is impossible to comment on the success or otherwise of mechanical ventilation in aiding survival (Table 2).

**Table 2:** Outcomes of mechanical ventilation in drowning

Study	Outcome measured	Outcome	Confounders
Fandell et al. [15]	Mortality Barotrauma	12/34 (35%) MV 6/12 (50%) died 9/12 (75%) pneumothorax, 8/12(66%) pneumomediastinum	Not controlled for other interventions
Petersen [19]	Mortality Pneumonia ARDS Barotrauma	7/72 (10%) died 10/72 (14%) barotrauma (all MV) 29/72 (40%) pneumonia 6/72 (9%) ARDS	Unclear number of MV patients; outcomes not group specific
Corbin [41]	Mortality	3/8 (38%) died	Not controlled for other interventions
Oakes et al. [17]	Mortality Barotrauma Pneumonia	25/40 (63%) MV 3/25 (12%) barotrauma 16/40 (40%) pneumonia 10/40 (25%) died	Not controlled for other interventions; outcomes not group specific
van Berkel et al. [36]	Mortality Pneumonia	25/102 (25%) MV 11/25 (52%) pneumonia (RR 17.3, P < 0.001) 6/25 (25%) died	Not controlled for other interventions
Lee [10]	Pneumonia	8/17 (47.1%) MV 0/17 (0.0%) pneumonia	Not controlled for other interventions; outcomes not group specific
al-Talafieh et al. [32]	Mortality Pneumonia Barotrauma	14/34 MV 5/34 (15%) died 6/34 (18%) pneumonia 1 PTX	Outcomes not group specific
Saidel-Odes et al. [52]	Mortality Pneumonia ARDS	11/69 (16%) MV patients 26/69 (38%) pneumonia 3/69 (4%) ARDS No deaths	Not controlled for other interventions; outcomes not group specific
Ballesteros et al. [38]	Mortality	21/43 (49%) MV 15/43 (35%) died	Not controlled for other interventions; outcomes not group specific
Kotsiou et al. [54]	Mortality ARDS	8/20 (40%) MV 0 deaths 8/20 (40%) mod/severe ARDS	Not controlled for other interventions ? Definition of ARDS
Michelet et al. [28]	Mortality Pneumonia Duration MV	30/88 (34%) MV 6/30 (20%) pneumonia 5/30 (17%) septic shock No deaths 3 ± 2 days	Not controlled for other interventions
Cerland et al. [39]	Mortality Pneumonia ARDS ECLS	64/144 (44%) MV 35/144 (24%) pneumonia 23/144 (16%) ARDS 45/144 (31%) died 2/64 (3%) ECLS	Not controlled for other interventions; outcomes not group specific
Michelet et al. [29]	Mortality Duration MV	40/76 (53) MV 15/76 (20%) died 8 (± 16) days	Unclear ventilatory modes and outcomes

MV, mechanical ventilation; ARDS, Acute Respiratory Distress Syndrome; ECLS, extra-corporeal life support

### Non-invasive ventilation

There were four papers [28, 29, 39, 40] reporting on non-invasive ventilation (NIV) with the majority (3/4, 75%) published since 2017 [28, 29, 39] (Table 3). The earliest report of successful use of NIV dates from 1982, where eleven patients were successfully treated with continuous positive airway pressure (CPAP) [40]. Recently, three larger studies all from France or its overseas territories have been published [28, 29, 39]. Cerland et al. report use of NIV in 28 patients with acute respiratory failure [39]. Outcomes are not explicitly reported, but all deaths (*n* = 45) in their cohort of 144 had experienced pre-hospital cardiac arrest [39]. The other two series of NIV patients are from the same group located in the south of France [28, 29]. Their 2017 paper describes a population of 25 patients who received NIV from emergency medical services (EMS) and additional 23 patients put on NIV in the ED after arrival at hospital. Four of the patients put on NIV by EMS subsequently received MV (three because of worsening respiratory failure), and all patients survived [28]. Patients receiving NIV were different from those receiving MV. They were more alert (Glasgow Coma Score 12 ± 3 NIV vs 7 ± 2 MV, P < 0.05) and were not as critically ill with lower Simplified Acute Physiology Scores (28 ± 8 vs 50 ± 19) and Sequential Organ Failure Assessment scores (2.4 ± 2 vs 6.5 ± 4) [28]. The authors noted a similar rate of improvement in oxygenation between NIV and MV after the first six hours [28]. The second paper retrospectively compared 38 matched pairs (*n* = 76) for fresh versus seawater drowning [29]. Thirteen patients received NIV and 40 patients MV. There were no reported failures of NIV [29]. The mean duration of treatment with NIV was 1.4 (± 2.4) days when the results are combined from both papers [28, 29] (Table 3).

**Table 3:** Outcomes of non-invasive ventilation in drowning

Study	Outcome measured	Outcome	Confounders
Modell et al. [16]	Mortality	24 spontaneously ventilating patients received intermittent positive end expiratory pressure 10/90 (11%) died	Not controlled for other interventions; outcomes not group specific
Dick et al. [40]	Mortality	11/18 (61%) NIV, 2/18 (11%) died	Not controlled for other interventions; outcomes not group specific
Cerland et al. [39]	Mortality Pneumonia ARDS	28/144 (19%) NIV 35/144 (24%) pneumonia 23/144 (16%) ARDS 45/144 (31%) died	Not controlled for other interventions; outcomes not group specific
Michelet et al. [28]	Mortality Conversion to MV Pneumonia Duration NIV	48/88 (55%) NIV 4/48 (8%) escalated to MV 1/48 (2%) pneumonia 1.4 ± 0.7 days	Not controlled for other interventions
Michelet et al. [29]	Mortality Pulmonary Complications Duration NIV	13/76 (17%) NIV 4/76 (5%) pneumonia 15/76 (20%) died 1.3 ± 5 days	Outcomes not group specific

NIV, non-invasive ventilation; ARDS, Acute Respiratory Distress Syndrome; MV, mechanical ventilation

### Hi-flow nasal prongs

There was a single article reporting on the use of HFNP [53]. Fifty-seven patients were treated with HFNP, and 12 were converted to MV for worsening ARDS with two patients ultimately requiring ECLS. There were two deaths in the series [53].

### Prophylactic antibiotics

Prophylactic antibiotics were used in 562 (28.5%) patients from 14 studies. Outcome data were only available on 311 patients from seven studies [9, 16, 33, 38, 41, 47, 52]. The mortality rate was 23/311 (7.4%). A single study [16] reported mortality in the patients that did not receive antibiotics (2/36, 5.6%). Two studies reported no improvement from the use of prophylactic antibiotics without including outcome data [18, 36] (Table 4).

**Table 4:** Outcomes of antibiotic prophylaxis

Study	Outcome measured	Outcome	Confounders
Modell et al. [16]	Mortality	54/90 (60%) ABP, 7/54 (13%) died 36/90 (40%) no ABP, 2/36 (6%) died	Not controlled for other interventions
Orlowski [18]	Unclear	“The use of corticosteroids, antibiotic prophylaxis and diuretics did not improve prognosis” (data not shown)	Not controlled for other interventions; outcomes not group specific
Corbin [41]	Mortality	79 patients treated. Minimum of one death, potentially three	Not controlled for other interventions; outcomes not group specific
Dick et al. [40]	Mortality	16/18 (89%) ABP 2/18 (11%) died	Not controlled for other interventions; outcomes not group specific
Oakes et al. [17]	Mortality Pneumonia	31/40 (78%) ABP 16/40 (40%) pneumonia 10/40 (25%) died	Not controlled for other interventions; outcomes not group specific
Simcock [33]	Mortality Pneumonia ARDS	68/121 (56%) ABP 12/68 (18%) died (1 from pneumonia, 1 from ARDS) 53/121 (44%) no ABP, 0 died	Not controlled for other interventions Treatment determined by severity of illness
van Berkel et al. [36]	Mortality Pneumonia Duration of MV	45/102 (44%) ABP 15/102(15%) pneumonia 7/102 (7%) died, 3 from pneumonia No effect of ABP on duration of MV, ICU LOS or hospital LOS (data not shown)	Not controlled for other interventions; outcomes not group specific
Lee [10]	Pneumonia	16/17 (94.1%) ABP 0/17 (0.0%) pneumonia	Not controlled for other interventions
Saidel-Odes et al. [52]	Mortality Pneumonia ARDS	42/69 (61%) ABP 26/69 (38%) pneumonia 3/69 (4%) ARDS No deaths	Not controlled for other interventions; outcomes not group specific
Gregorakos et al. [9]	Pneumonia ARDS	43/43 ABP 4/43 (9%) pneumonia 1/43 (2%) died from pneumonia	Not controlled for other interventions
Ballesteros et al. [38]	Mortality Septic outcomes	27/43 (62.8%) ABP 15/43 (35%) died 1/43 (2%) died from pneumonia No effect of ABP on outcomes (data not shown)	Not controlled for other interventions; outcomes not group specific
Kim [47]	Mortality	9/9 (100%) ABP 2/9 (22.2%) died	All patients received ECLS
Cerland et al. [39]	Mortality Pneumonia ARDS	85/144 (59%) ABP 35/144 (24%) pneumonia 23/144 (16%) ARDS 45/144 (31%) died	Not controlled for other interventions; outcomes not group specific
Robert et al. [30]	Mortality Pneumonia ARDS	44/74 (59%) ABP 36/74 (49%) pneumonia 25/74 (34%) ARDS 19/74 (26%) died	Outcomes not group specific

ABP, antibiotic prophylaxis; ARDS, Acute Respiratory Distress Syndrome; ECLS, extracorporeal life support

### Prophylactic steroids

The outcomes reported from seven papers for prophylactic steroids showed no benefit [16, 18, 33, 36, 40, 41, 52]. Overall, 264 patients received prophylactic steroids and 31 (11.7%) died. Ninety-one patients were reported as not being treated with prophylactic steroids and two died (2.2%). One paper reported an increased hospital length of stay (LOS) in patients who received steroids but did not require mechanical ventilation compared with those not receiving steroids (3.2 vs 1.7 days, supporting data not presented) [41]. A second paper described performing regression analysis to measure the effect of steroids [36] and concluded that there was no effect, but no data supporting this were included [36].

### Prophylactic diuretics

Prophylactic use of diuretics has been reported as having no clinical benefit [18, 40, 41]. However, pre-hospital use of forced diuresis with frusemide by EMS is described in a series of 69 drowning patients from the Dead Sea [52]. There were no fatalities in this study [52]. Similar results are reported in a series of 43 patients from Greece, where only two patients required escalation of therapy secondary to respiratory compromise [9].

### Other treatment modalities

One paper reports the use of bronchodilators (aminophylline) in 22/98 patients with a minimum of one and potentially two deaths, as well as the use of plasma in 12/98 patients with a minimum of one and potentially three deaths [41]. Mortality results were extrapolated from grouped data.

## Discussion

The key finding from this review is the lack of evidence informing the treatment of the lung injuries associated with drowning. There was a single retrospective comparison of treatments [28]. This is in stark contrast to eight included studies [10, 15, 16, 18, 29, 36, 37, 41] that compare outcomes of drownings between fresh and salt water.

In 1973, it was reported that the lung injury associated with drowning was rapidly reversible with the application of positive pressure mechanical ventilation [56]. Following this, several studies reported a high incidence of barotrauma when treating drowning with MV [15, 17, 19]. Subsequently, the similarities between the lung injury in drowning and ARDS have been established [6]. Randomised trials have established the safety and efficacy of LPV in ARDS [57], and it has been adopted as best practice in the management of the lung injury associated with drowning [7, 11]. This may explain the decrease in reported barotrauma associated with MV in recent studies; however, it was not documented as a measured outcome [28, 29, 39].

The use of NIV in drowning was first reported in 1982 [40]; however, it has only been recently that any substantive evidence has been presented regarding the efficacy of NIV in the drowning patient [28, 29, 39]. When compared with MV for the treatment of drowning patients, NIV is similarly effective as MV in reversing hypoxia but is required for a significantly shorter duration (1.4 ± 2.4 vs 6 ± 12 days, *P* = 0.004) [28, 29]. This has to be interpreted with caution given different indications for both treatments [15, 32, 39, 52, 54], but a recent study also established the efficacy and safety of NIV in mild to moderate ARDS [58].

Oxygen therapy using HFNP has been adopted widely from the treatment of bronchiolitis to many other causes of respiratory insufficiency in children and adult patients [59]. There is a single report of its use in drowning [53]. There is, however, such a lack of evidence that we can only recommend clinical judgement be applied when deciding on therapeutic strategies when supplemental oxygen alone is insufficient.

It was surprising that the majority of published studies regarding the treatment of drowning are on the use of ECLS. The Extracorporeal Life Support Organization international database has over 400 centres contributing data [21]. Despite this, there were only 247 drowning patients included over a 30-year period. Clearly, the use of ECLS in drowning patients is not a common occurrence. However, the survival rates in patients with cardiac arrest (57% with ROSC, 23% with ECLS) compare favourably to published survival rates post-drowning associated cardiac arrest in Germany (18%) [60], Sweden (14%) [61] and France (9%) [62]. There were one meta-analysis and one systematic review examining ECLS in the treatment of drowning and hypothermia that were not included in this paper [63, 64]. Both studies grouped drowning and avalanche patients in ‘asphyxial’ groups, and the drowning outcomes could not be separated. However, the outcomes for this group of patients were much worse when compared with isolated hypothermic cardiac arrest with 23.4% vs 67.7% survival [63] and an odds ratio for survival of 0.19 (0.11–0.35) [64].

No study reported on the efficacy of any of the adjuvant therapies in isolation. Added to this was the confounder that all treatments were administered at the clinicians’ discretion and, almost certainly, there was significant treatment bias with sicker patients more likely to receive MV and adjuvant therapies, such as steroids and antibiotics. van Berkel et al. (1996) did conduct a regression analysis attempting to control for confounding variables and concluded that there was no benefit from any of the adjuvant therapies with regards to duration of MV, hospital or ICU LOS [36]. They also concluded that MV was a risk factor for developing pneumonia post-drowning without presenting the data or outlining the variables included in the analysis [36]. Clinical trials in ARDS have established a lack of efficacy for steroids [65] and surfactant [66, 67]. The evidence for or against diuretic therapy in ARDS is less clear [68, 69]. The lack of evidence in drowning prevents any recommendations.

## Implications for future research

While drowning is a common cause of death worldwide [1], it is neither a common cause of ED presentations [70, 71] nor hospital admissions [72]. This may explain the apparent lack of evidence regarding its management. The lack of comparative studies and scarcity of multi-centre collaborations are of concern and must be addressed urgently. This is especially so given the demonstrated value of the Extracorporeal Life Support Organization’s international registry in informing the use of ECMO in drowning patients.

### Limitations

This structured evidence-based review was aimed at establishing the primary evidence behind the treatment of the lung injuries associated with drowning. The World Health Organisation published a uniform definition of drowning and its outcomes in 2005 [4]. More than half of the studies included in this review were published after 2005. Unfortunately, only four of them use the correct definition [28–30, 50]. Without a consistent definition of a drowning patient, it is hard to integrate the published evidence.

The search strategy excluded any papers written in languages other than English. Given the very low quality of evidence, one or two high-quality non-English language papers may have changed the findings of the review.

## Conclusions

There is a dire lack of evidence informing the management of the drowning patient. This makes any recommendations regarding best practice impossible other than to follow local guidelines and clinical judgement. There is an urgent need for high-quality research on the treatment of drowning. Duration of immersion is a critical factor in patient prognosis [8], and as such, prevention is currently the most effective strategy in reducing drowning mortality.

## Supplementary Information

**Additional file 1**. Medline search strategy.

**Additional file 2**. GRADE evidence summary of included studies.

**Additional file 3**. Summary Table of included studies.

## Data Availability

All data generated or analysed in this study are included in this published article (and its Additional files).

## Abbreviations

ABP: Antibiotic prophylaxis
ARDS: Acute respiratory distress syndrome
CPC: Cerebral performance category
ECLS: Extra-corporeal life support
ECPR: ECLS-assisted cardio-pulmonary resuscitation
EMS: Emergency medical services
GNO: Good neurological outcome
GOSE: Glasgow outcome scale extended
GRADE: Grading of recommendations assessment, development and evaluation methodology
HFNP: High flow nasal prongs
ICU: Intensive care unit
LOS: Length of stay
LPV: Lung protective ventilation
MV: Mechanical ventilation,
NIV: Non-invasive ventilation
PCPC: Paediatric cerebral performance category
PRISMA: Preferred reporting items for systematic reviews and meta-analyses
ROSC: Return of spontaneous circulation
USFD: Utstein style for drowning
