# Supplementary material for: Treatment of the lung injury of drowning: a systematic review
Source: Crit Care. 2021 Jul 19;25:253. doi: 10.1186/s13054-021-03687-2 (PMC8287554; doi:10.1186/s13054-021-03687-2)
Supplement: Supplementary file 1 — Additional file 1. Medline search strategy. [file 13054_2021_3687_MOESM1_ESM.docx]

Additional File 1.

**Search Strategy (Medline)**

*Environment*

“critical care” OR “intensive care”

“emergency care” OR “emergency health service” OR “emergency health services” OR “emergency medical service” OR “emergency medical services” OR “emergicenter” OR “emergicenters” OR “medical emergency service” OR “medical emergency services” OR “prehospital emergency care”

“emergency medicine” OR “accident and emergency department” OR “emergency department” OR ”emergency departments” OR “emergency hospital service” OR “emergency hospital services” OR “emergency outpatient unit” OR “emergency outpatient units” OR “emergency room” OR “emergency rooms” OR “emergency unit” OR “emergency ward” OR “emergency wards” OR “hospital emergency service” OR “hospital emergency services” OR “hospital service emergencies”

*Condition*

"drowning"

"near drowning"

*Intervention*

“non invasive ventilation” OR “non invasive ventilations” OR “non-invasive ventilation” OR “non-invasive ventilations” OR “noninvasive ventilation” OR “noninvasive ventilations” OR “biphasic intermittent positive airway pressure” OR “ippv” OR “inspiratory positive pressure ventilation” OR “inspiratory positive-pressure ventilation” OR “intermittent positive pressure ventilation” OR “intermittent positive-pressure ventilation” OR “artificial respiration” OR “artificial respirations” OR “mechanical ventilation” OR “mechanical ventilations” OR “acute respiratory distress syndrome” OR “adult respiratory distress syndrome” OR “human ards” OR “shock lung”

“ecls treatment” OR “ecls treatments” OR “ECLS treatment” OR “ECLS treatments” OR “extracorporeal life support” OR “extracorporeal life supports” OR “extracorporeal membrane oxygenation” OR “extracorporeal membrane oxygenations” OR “life support, extracorporeal” OR “life supports, extracorporeal” OR “membrane oxygenation, extracorporeal” OR “membrane oxygenations, extracorporeal” OR “oxygenation, extracorporeal membrane” OR “oxygenations, extracorporeal membrane” OR “support, extracorporeal life” OR “supports, extracorporeal life” OR “treatment, ecls” OR “treatment, ECLS” OR “treatments, ecls” OR “treatments, ECLS”

“anti-bacterial agents” OR “agents, anti-bacterial” OR “agents anti-mycobacterial” OR “agents, antibacterial” OR “agents, antimycobacterial” OR “agents, bactericidal” OR “anti bacterial agents” OR “anti bacterial compounds” OR “anti mycobacterial agents” OR “anti-bacterial agents” OR “anti-bacterial compounds” OR “anti-mycobacterial agents” OR “antibacterial agents” OR “antibiotic” OR “antibiotics” OR “antimycobacterial agents” OR “bacteriocidal agents” OR “bacteriocides” OR “compounds, anti-bacterial”

“adrenal cortex hormones” OR “corticoids” OR “corticosteroids” OR “hormones, adrenal cortex”

“diuretics” OR “diuretic” OR “diuretic effect” OR “diuretic effects” OR “effect, diuretic” OR “effects, diuretic”
