# Supplementary material for: Treatment of the lung injury of drowning: a systematic review
Source: Crit Care. 2021 Jul 19;25:253. doi: 10.1186/s13054-021-03687-2 (PMC8287554; doi:10.1186/s13054-021-03687-2)
Supplement: Supplementary file 2 — Additional file 2. GRADE evidence summary of included studies. [file 13054_2021_3687_MOESM2_ESM.docx]

Additional File 3.

**GRADE Evidence Profile**

| Study | Method | Bias | Inconsistency | Imprecision | Publication Bias | Evidence Quality |
| --- | --- | --- | --- | --- | --- | --- |
| Al-Talafieh 1999 | Retrospective case series | Very serious | No serious inconsistency | No serious imprecision | Strongly suspected | Very low |
| Ballesteros 2009 | Retrospective case series | Serious | No serious inconsistency | Serious | Strongly suspected | Very low |
| Bauman 2019 | Retrospective case series | Very serious | Serious | No serious imprecision | Strongly suspected | Very low |
| Burke 2016 | Registry database analysis | Very serious | No serious inconsistency | No serious imprecision | Strongly suspected | Very low |
| Cerland 2018 | Retrospective case series | Very serious | No serious inconsistency | No serious imprecision | Strongly suspected | Very low |
| Champigneulle 2015 | Retrospective case series | Very serious | Serious | No serious imprecision | Strongly suspected | Very low |
| Corbin 1981 | Retrospective case series | Very serious | No serious inconsistency | No serious imprecision | Strongly suspected | Very low |
| Coskun 2010 | Retrospective case series | Very serious | Serious | No serious imprecision | Strongly suspected | Very low |
| Dick 1982 | Retrospective case series | Very serious | No serious inconsistency | No serious imprecision | Strongly suspected | Very low |
| Eich 2007 | Retrospective case series | Very serious | Serious | No serious imprecision | Strongly suspected | Very low |
| Fandel 1976 | Retrospective case series | Very serious | No serious inconsistency | No serious imprecision | Strongly suspected | Very low |
| Farstad 2001 | Retrospective case series | Very serious | Very serious | No serious imprecision | Strongly suspected | Very low |
| Gregorakos 2009 | Retrospective case series | Very serious | No serious inconsistency | No serious imprecision | Strongly suspected | Very low |
| Khorsandi 2017 | Retrospective  case series | Very serious | Very serious | No serious imprecision | Strongly suspected | Very low |
| Kim 2014 | Retrospective case series | Very serious | Serious | No serious imprecision | Strongly suspected | Very low |
| Kim 2019 | Retrospective case series | Very serious | No serious inconsistency | No serious imprecision | Strongly suspected | Very low |
| Kotsiou 2014 | Retrospective case series | Very serious | No serious inconsistency | No serious imprecision | Strongly suspected | Very low |
| Lee 1998 | Retrospective case series | Very serious | No serious inconsistency | No serious imprecision | Strongly suspected | Very low |
| Lee 2020 | Retrospective case series | Very serious | Very serious | No serious imprecision | Strongly suspected | Very low |
| Mair 1994 | Retrospective case series | Very serious | Very serious | No serious imprecision | Strongly suspected | Very low |
| Michelet 2017 | Retrospective case series | Serious | No serious inconsistency | No serious imprecision | Strongly suspected | Very low |
| Michelet 2019 | Retrospective cohort | Serious | No serious inconsistency | No serious imprecision | Strongly suspected | Very low |
| Modell 1976 | Retrospective case series | Very serious | No serious inconsistency | No serious imprecision | Strongly suspected | Very low |
| Oakes 1982 | Retrospective case series | Very serious | No serious inconsistency | No serious imprecision | Strongly suspected | Very low |
| Petersen 1977 | Retrospective case series | Very serious | No serious inconsistency | No serious imprecision | Strongly suspected | Very low |
| Robert 2017 | Retrospective case series | Very serious | No serious inconsistency | No serious imprecision | Strongly suspected | Very low |
| Saidel-Odes 2003 | Retrospective case series | Very serious | Serious | No serious imprecision | Strongly suspected | Very low |
| Saltiel 1989 | Retrospective case series | Very serious | Serious | No serious imprecision | Strongly suspected | Very low |
| Scaife 2007 | Retrospective case series | Very serious | Very serious | No serious imprecision | Strongly suspected | Very low |
| Simcock 1986 | Retrospective case series | Very serious | No serious inconsistency | No serious imprecision | Strongly suspected | Very low |
| Skarda 2012 | Retrospective case series | Very serious | Very serious | No serious imprecision | Strongly suspected | Very low |
| Steiner 1991 | Retrospective case series | Very serious | Very serious | No serious imprecision | Strongly suspected | Very low |
| Suominen 2010 | Retrospective case series | Very serious | Serious | No serious imprecision | Strongly suspected | Very low |
| van Berkel 1996 | Retrospective case series | Very serious | No serious inconsistency | No serious imprecision | Strongly suspected | Very low |
| Walpoth 1990 | Retrospective case series | Very serious | Very serious | No serious imprecision | Strongly suspected | Very low |
| Wanscher 2012 | Retrospective case series | Very serious | Very serious | No serious imprecision | Strongly suspected | Very low |
| Watson 2017 | Registry database analysis | Very serious | Very serious | No serious imprecision | Strongly suspected | Very low |
| Weber 1998 | Retrospective case series | Very serious | Very serious | No serious imprecision | Strongly suspected | Very low |
| Weuster 2016 | Retrospective case series | Very serious | Very serious | No serious imprecision | Strongly suspected | Very low |
| Wollenek 2002 | Retrospective case series | Very serious | Very serious | No serious imprecision | Strongly suspected | Very low |
