# Supplementary material for: Treatment of the lung injury of drowning: a systematic review
Source: Crit Care. 2021 Jul 19;25:253. doi: 10.1186/s13054-021-03687-2 (PMC8287554; doi:10.1186/s13054-021-03687-2)
Supplement: Supplementary file 3 — Additional file 3. Summary Table of included studies. [file 13054_2021_3687_MOESM3_ESM.docx]

Additional File 3. Treatment strategies reported for the lung injury associated with drowning.

| Study | Country | Study Aims | N  Pop  Setting | Treatment options | | | | | | | Methodology/  GRADE level of evidence(12) | Comments |
| --- | --- | --- | --- | --- | --- | --- | --- | --- | --- | --- | --- | --- |
|  |  |  |  | Antibiotics | Steroids | Diuretic | NIV | HFNC | MV | ECLS |  |  |
| Fandell et al 1976(14) | United States of America | Evaluate the clinical aspects of near-drowning in the paediatric age group | 32  Children  Inpatient |  |  |  |  |  | X |  | Retrospective case series  Very low | 12/32 (37.5%) MV for decreased GCS;  9/12 (75%) complicated by pneumothorax |
| Modell et al 1976(15) | United States of America | Describe the clinical course and pathophysiology of drowning patients | 91  Adult + Children  ED + ICU | X | X |  |  |  |  |  | Retrospective case series  Very low | No apparent benefit for patients given antibiotics and steroids |
| Petersen 1977(18) | United States of America | Quantify the morbidity of near drowning in children and determine if current practice can be improved | 72  Children  ED |  |  |  |  |  | X |  | Retrospective case series  Very low | 6/72 (08.3%) complicated by ARDS;  29/72 (40.3%) aspiration pneumonia;  7/72 (09.7%) pneumothorax |
| Orlowski 1979(17) | Australia  United States of America  India | Report 93 cases of post-submersion syndrome in the paediatric age group and discus prognostic factors | 93  Children  Inpatient | X | X | X |  |  |  |  | Retrospective case series  Very low | 4/30 (13.3%) with normal chest x-ray died; use of steroids, antibiotics or frusemide did not improve prognosis (no data given) |
| Corbin 1981(42) | Barbados | Review the impact of increased tourism on drowning presentations to hospital and outcomes | 98  Adult + Children  Inpatient | X | X | X |  |  | X |  | Retrospective case series  Very low | 4/98 (04.1%) deaths  Treatment with steroids associated with increased hospital LOS in non-ventilated patients  Reports use of bronchodilator and plasma for treatment of drowning |
| Dick et al 1982(41) | South Africa | Describe the early clinical signs and management of “secondary drowning” | 18  Adult + Children  ICU | X | X | X | X |  |  |  | Retrospective case series  Very low | First case series of NIV (11 /18 (61.1%) CPAP);  focus on ‘secondary’ drowning |
| Oakes et al 1982(16) | United States of America | Identify prognostic criteria and evaluate methods of cerebral resuscitation in drowning patients | 40  Adult + Children  Inpatient | X |  |  |  |  | X |  | Retrospective case series  Very low | 16/31 (51.6%) given prophylactic antibiotics developed pneumonia  3/25 (12.0%) MV complicated by pneumothorax  7/16 (43.8%) patients with normal chest x-ray required MV |
| Simcock 1986(32) | United Kingdom | Review the management and clinical course of patients after rescue from drowning | 123  Adult + Children  ED | X | X |  |  |  |  |  | Retrospective case series  Very low | Rapid resolution of respiratory difficulties |
| Saltiel et al 1989 (21) | United States of America | Report on the use of cardiopulmonary bypass to rewarm three victims of prolonged cold water submersion | 3  Adult+  Children |  |  |  |  |  |  | X | Retrospective case series  Very low | 1/3 (33.3%) died  2/3 (66.6%) survived  1/3 (33.3%) good neurological outcome  1/3 (33.3%) poor neurological outcome |
| Walpoth et al 1990 (33) | Switzerland | Focus on our experience with extracorporeal blood rewarming in patients with deep hypothermia and cardiopulmonary arrest | 2  Adult |  |  |  |  |  |  | X | Retrospective case series  Very low | 0/2 (0.0%) survived |
| Steiner et al 1991 (24) | United States of America | Retrospective review of paediatric trauma patients treated with ECLS for respiratory failure | 8 Children |  |  |  |  |  |  | X | Retrospective case series  Very low | 3/8 (37.5%) survived with good neurological outcomes |
| Mair et al 1994 (49) | Austria | Investigate if plasma potassium, pH and activated clotting time predict outcome in patients with severe hypothermia and circulatory arrest | 7  Adult + Children |  |  |  |  |  |  | X | Retrospective case series  Very low | 1/7 (14.2%) survived |
| van Berkel et al 1996(35) | Netherlands | Determine criteria to identify at the time of admission drowning patients likely to develop lung injury | 125  Adult + Children  ICU | X | X |  |  |  |  |  | Retrospective case series  Very low | Occurrence of pneumonia not affected by prophylaxis |
| Lee 1998 (9) | Singapore | Examine the physiologic responses in fresh and seawater drownings and the outcomes | 17  Adult + Children | X |  |  |  |  | X |  | Retrospective case series  Very low | 14/17 (82.3%) survived  16/17 (94.1%) had PaO_2_/FiO_2_ < 300 mm Hg  17/17 (100%) pulmonary oedema on chest x-ray |
| Weber et al 1998 (26) | United States of America | Examine whether ECLS might improve survival | 4 Children |  |  |  |  |  |  | X | Retrospective case series  Very low | 1/4 survived  1/4 good neurological outcome |
| al-Talafieh et al 1999(31) | Jordan | Evaluate 34 cases of drowning for clinical features | 34  Adult + Children  ED |  |  |  |  |  | X |  | Prospective case series  Very low | 14 patients put on MV for variety of reasons; all survived |
| Farstad et al 2001 (46) | Norway | Present a retrospective analysis of patietns with hypothermia and cardiorespiratory arrest or cardiorespiratory insufficiency rewarmed by extracorporeal circulation | 14  Adult + Children |  |  |  |  |  |  | X | Retrospective case series  Very low | 1/14 (07.1%) survived  1/14 (07.1) poor neurological outcome |
| Wollenek et al 2002 (52) | Austria | Describe experience with three paediatric patients after cold water submersion | 3  Children |  |  |  |  |  |  | X | Retrospective case series  Very low | 2/3 (66.6%) survived  1/3 (33.3%) good neurological outcome  1/3 (33.3%)poor neurological outcome |
| Saidel-Odes et al 2003(53) | Israel | Assess the clinical spectrum of “near-drowning in the Dead Sea syndrome” and to evaluate current trends in management and outcome | 69  Adult  Inpatient | X | X | X |  |  |  |  | Retrospective case series  Very low | Dead Sea; 26/42 given antibiotics got pneumonia |
| Eich et al 2007 (45) | Germany | Report on drowned children who underwent attempted resuscitation on cardiopulmonary bypass | 12  Children |  |  |  |  |  |  | X | Retrospective case series  Very low | 5/12 (41.7%) survived  2/12 (16.7%) good neurological outcome  3/12 (25.0%) poor neurological outcome |
| Scaife et al 2007 (22) | United States of America | Present experience with a pre-emptive strategy for extreme hypothermic arrest | 4  Children |  |  |  |  |  |  | X | Retrospective case series  Very low | 2/4 (50%) survived |
| Gregorakos et al 2009(8) | Greece | Report experience with hospitalized victims of submersion in warm seawater, who survived for at least 24 hours after the accident, with special emphasis on the respiratory consequences of submersion | 43  Adult  Inpatient | X |  | X | X |  |  |  | Retrospective case series  Very low | 17 patients with ALI; 15 patients with ARDS – 1 treated with MV; one treated with NIV; the rest were treated with supplemental oxygen |
| Ballesteros et al 2009(39) | Spain | Evaluate the profile of near-drowning population and the influence of several clinical variables on outcome in pts admitted to ICU  All pts had received CPR | 43  Adult + Children  ICU | X |  |  |  |  | X |  | Retrospective case series  Very low | MV used on patients intubated pre-hospital; prophylactic antibiotics had no influence on outcome (data not shown) |
| Coskun et al 2010 (44) | Germany | Analysis of data after rewarming by extra-corporeal circulation in drowning and near drowning with severe hypothermia | 13  Children |  |  |  |  |  |  | X | Retrospective case series  Very low | 5/13 (38.4%) survived  1/13 (07.7%) good neurological outcome  1/13 (07.7%) mild neurological deficit  3/13 (23.1%) severe neurological deficit |
| Suominen et al 2010 (50) | Finland | Present retrospective single centre data on hypothermic submerged children treated with cardiopulmonary bypass | 9  Children |  |  |  |  |  |  | X | Retrospective case series  Very low | 1/9 (11.1%) long term survivor  1/9 (11.1) mild neurological deficit |
| Skarda et al 2012 (23) | United States of America | Report on use of extracorporeal cardiopulmonary resuscitation for treatment of hypothermic cardiac arrest | 7  Children |  |  |  |  |  |  | X | Retrospective case series  Very low | 0/7 (0%) survived |
| Wanscher et al 2012 (34) | Denmark | Compare two groups (with and without circulatory arrest) and assess outcome in profoundly hypothermic victims | 7  Children |  |  |  |  |  |  | X | Retrospective case series  Very low | 7/7 (100%) survived  4/7 (57.1%) GCS 15 at discharge  1/7 (14.2%) able to return to school |
| Kim et al 2014 (48) | Republic of Korea | Present the early outcomes of ECLS used for near-drowning patients | 9  Adult  Children | X |  |  |  |  |  | X | Retrospective case series  Very low | 7/9 survived with good neurological outcome  No ECLS complications |
| Kotsiou et al 2014 (38) | Greece | Present a descriptive, retrospective study of drowning or near drowning victims admitted to the ED | 27  Adult  ED |  |  |  |  |  | X |  | Retrospective case series  Very low | 12 mild; 7 moderate and 1 severe ARDS; supplemental oxygen for mild; immediate MV for moderate/severe |
| Champigneulle et al 2015 (30) | France | Describe cohort of refractory hypothermic cardiac arrest due to drowning treated with ECLS | 20  Adults | X |  |  |  |  |  | X | Retrospective case series  Very low | 2/20 (10%) 6-month survival 1/20 (5%) good neurological outcome |
| Burke et al 2016 (20) | Not reported | Characterize survival rates and risk factors for death in drowning victims who receive ECLS | 247  Adult + Children  ELSO registry |  |  |  |  |  |  | X | Extracorporeal Life Support Organisation international registry data  Very low | 30 years of data – use of ECLS for drowning still an uncommon event.  127/247 survived |
| Khorsandi et al 2016 (47) | United Kindom | Outcomes of accidental hypothermic cardiac arrest treated with ECLS | 2  Adults |  |  |  |  |  |  | X | Retrospective case series  Very low | 1/2 died  Complicated by overdoses |
| Weuster et al 2016 (51) | Germany | describe our experience in nine individual patients with AH after drowning | 9  Children  Adults |  |  |  |  |  |  | X | Retrospective case series  Very low | 2/9 (22%) survived  7/8 who suffered cardiac arrest died  1/1 with ARDS survived |
| Cerland et al 2017(40) | French West Indies | Describe the frequency, nature, and consequences of near-drowning-related pneumonia | 144  Adult + Children  ICU | X |  |  | X |  |  |  | Retrospective case series  Very low | 77 patients ARF; 23 patients ARDS; 28 patients NIV; 64 patients MV; 2 patients ECLS;  prophylactic antibiotics in 85; pneumonia in 35 |
| Michelet et al 2017(27) | France | Analyse the clinical course of drowning related ARF patients both in the prehospital and the ICU settings and to describe the efficacy of NIV when used. | 126  Adult  ICU |  |  |  | X |  |  |  | Retrospective case series  Very low | NIV duration 1-2 days (BiPAP and CPAP); MV a risk factor for pneumonia |
| Robert et al 2017(29) | France | Describe the bacterial species and their antibiotic susceptibility from seawater drowning patients in the Mediterranean Sea | 74  Adult  ICU | X |  |  |  |  |  |  | Retrospective case series  Very low | 44/75 pts got antibiotics within first 48 hrs; 36/44 developed pneumonia;  25/74 pts developed ARDS |
| Watson et al 2017 (25) | United States of America | Describe recent experience with ECLS in critically injured children | 4 children |  |  |  |  |  |  | X | National trauma database  Very low | 4 cases in 5 years  3/4 survived |
| Bauman et al 2019 (19) | United States of America | Characterise experience treating of multiple pediatric hypothermic cardiac arrest victims | 3  Children |  |  |  |  |  |  | X | Retrospective case series  Very low | 2/3 survived  1/3 good neurological outcome |
| Michelet et al 2019(28) | France | Compare the respiratory function of ARF patients after unintentional drowning occurring in sea water and fresh water | 242  Adult  ICU |  |  |  | X |  | X |  | Retrospective cohort study  Very low | NIV used for 13 patients, duration 1-2 days; MV used for 21 patients, duration7-9 days; no difference in outcomes between fresh and seawater. |
| Kim et al 2019(37) | Republic of Korea | Investigate the effect of high flow nasal prongs in the treatment of moderate ARDS caused by drowning | 57  Adult ED |  |  |  |  | X |  |  | Retrospective case series  Very low | Moderate ARDS post drowning; 45 patients; treatment was successful; 12 converted to MV; 2 ECLS |
| Lee et al 2020 (36) | Republic of Korea | Evaluate experiences and outcomes with ECLS support in patients with traumatic lung injury | 18  Adult |  |  |  |  |  |  | X | Retrospective case series  Very low | 15/18 (83% survival) |

AB = antibiotic prophylaxis, NIV = non-invasive ventilation, HFNC = high flow nasal prongs, MV = mechanical ventilation, ECLS = extracorporeal life support. Adult > 17 years of age, Child ≤ 17 years of age, ARDS = acute respiratory distress syndrome, ARF = acute respiratory failure, BiPAP = bilevel positive airway pressure, CPAP = continuous positive pressure airway pressure, ED = emergency department, ICU = intensive care unit, inpatient = participants admitted to hospital, LOS = length of stay (days).
